# Electrochemical detection of different p53 conformations by using nanostructured surfaces

**DOI:** 10.1038/s41598-019-53994-6

**Published:** 2019-11-22

**Authors:** Sarah Tonello, Francesca Stradolini, Giulia Abate, Daniela Uberti, Mauro Serpelloni, Sandro Carrara, Emilio Sardini

**Affiliations:** 1 Department of Information Engineering, University of Brescia, Brescia, Italy; 2 Integrated Systems Laboratory (LSI), EPFL, Lausanne, Switzerland; 3 Department of Molecular and Translational Medicine, University of Brescia, Brescia, Italy

**Keywords:** Assay systems, Biomarkers, Metalloproteins, Characterization and analytical techniques

## Abstract

Protein electrochemistry represents a powerful technique for investigating the function and structure of proteins. Currently available biochemical assays provide limited information related to the conformational state of proteins and high costs. This work provides novel insights into the electrochemical investigation of the metalloprotein p53 and its redox products using label-free direct electrochemistry and label-based antibody-specific approaches. First, the redox activities of different p53 redox products were qualitatively investigated on carbon-based electrodes. Then, focusing on the open p53 isoform (*denatured p53*), a quantitative analysis was performed, comparing the performances of different bulk and nanostructured materials (carbon and platinum). Overall, four different p53 products could be successfully discriminated, from wild type to denatured. Label-free analysis suggested a single electron exchange with electron transfer rate constants on the order of 1 s^−1^. Label-based analysis showed decreasing affinity of pAb240 towards denatured, oxidized and nitrated p53. Furthermore, platinum nanostructured electrodes showed the highest enhancement of the limit of detection in the quantitative analysis (100 ng/ml). Overall, the obtained results represent a first step towards the implementation of highly requested complex integrated devices for clinical practices, with the aim to go beyond simple protein quantification.

## Introduction

Electrochemistry represents a powerful technique for investigating protein concentration and conformation in biological samples^1^. In recent decades, screen-printed electrodes (SPEs) have been widely studied as a promising strategy for protein investigation. These on-chip electrochemical cells host the three electrodes necessary to satisfy a complete electrochemical cell (*reference*, *working* and *counter electrodes* - RE, WE and CE, respectively) on a small surface, leading to a considerable improvement in terms of portability, ease of customization, reduction of sample volumes and rapid integration into complex devices for long-term and real-time measurements^2^. Regarding the sensitivity of this approach, nanostructures represent a powerful tool for exploiting the potentiality of SPE-based bioelectrochemistry^3^. Their nanosized structures allow for a high increase in the surface-to-volume ratio of any electrode, thus augmenting the space available for interacting with biomolecules^4^. Furthermore, nanostructure electronic properties ensure direct electron transfer from the target biomolecules to the electrodes^5^. These properties contribute to enhancing the sensitivity to small changes in biomolecule concentration or conformation, particularly for early disease-related biomarker detection up to the subnanomolar range^6,7^. Among organic nanostructures, carbon is the most adopted material for the design of customized nanostructured biosensors, with a wide variety of structures and deposition methods (including electrochemical growth or drop-casting)^8^. Among inorganic nanostructures, despite metals such as gold and silver being the most widely used, platinum (Pt) nanoparticles have attracted attention for the design of nanostructured biointerfaces for biological applications. Their high surface area, high electrocatalytic efficiency and possibility to customize the electrodeposition with other materials could ensure unique properties in terms of electrochemical analysis^9^. Unlike carbon-based nanostructures, metals are manufactured more frequently using other deposition techniques (electrochemical growth or *chemical vapor deposition* (CVD)^10^) with a very high level of standardization and shape-customization^11^. Considering this scenario, the combination of different nanostructures and electrochemical techniques is a valuable tool to obtain sensitive feedback about proteins in biological samples. Specifically, the study of protein structures and chemical modifications could provide new insights into the complexity of their activities (e.g., redox regulation)^12^ due to the strong correlation between protein conformational dynamics and their functions.

In this regard, the p53 protein represents an attractive metallo-redox sensitive protein, which has been widely investigated for its involvement in different pathophysiological processes. p53 is located at the crossroads of complex networks of stress response pathways, with a crucial effect on the cellular fate^13^. The inactivation of the p53 tumor suppressor is a frequent event in tumorigenesis found in human cancers that can also be due to the transition from the wild-type conformation to a mutant conformation. Thus, the possibility of recognizing several mutant p53 conformations and understand their functions could help in the development of new personalized therapeutic approaches that are useful in a broad range of human cancers^14^. Several studies have demonstrated that conformationally altered p53 could also occur in the absence of mutations^15^, and recently, this denatured p53 isoform was found to be possibly implicated in the onset of neurodegenerative diseases^16–19^. As a metalloprotein, it is extremely attractive since its electron transfer properties can be easily recorded by means of a direct electrochemistry approach, and the loss/gain of specific conductive groups can be correlated with specific protein conformational modifications^20^.

In light of these findings, the possibility of discriminating among different conformations of p53 might lead to a significant improvement in clinical applications, since these structural changes may be related to specific losses or gains of function. To date, only a few studies have investigated p53 conformations using direct electrochemistry. The native p53 structure highlights small current peaks due to tryptophan and tyrosine^21^, while an open isoform, probably due to loss of the zinc atom, shows an enhancement of the electrochemical response^20^. To our knowledge, no studies addressing the discrimination among different redox states of p53 protein have been performed. Thus, this paper introduces novel insights into the electrochemical investigation of the p53 conformation and its redox products using label-free direct electrochemistry and label-based antibody-specific approaches. The surfaces of the carbon (C) and platinum (Pt) electrodes were characterized by means of both electrochemical and microscopy techniques. The redox activity of different p53 redox products was investigated first on carbon-based electrodes, and then the performances of different bulk and nanostructured materials (C and Pt) were compared, focusing on the quantification of the completely opened isoform of p53 (*denatured* p53).

## Materials and Methods

### Materials

SPEs (model for carbon, DRP-C110; model for platinum, DRP-Pt550) were purchased from DropSens (Spain). Electrodes consisted of a 0.12 cm^2^ C/Pt WE, a silver (Ag) RE and a C/Pt CE. *Multi-walled carbon nanotube* (MWCNT, diameter: 10 nm; length: 1–2 µm, 90% purity, DropSens (Spain)) powder was dispersed in chloroform to a concentration of 2 mg/ml and then homogenized by sonication for 1 h. All other chemicals were purchased from Sigma Aldrich. Last, wild-type p53 recombinant protein was purchased from Santa Cruz Biotechnology Inc., Heidelberg (Germany).

### Electrode modifications with nanostructures

Nanostructure-modified C-SPEs were prepared by a drop-casting technique^22^. Briefly, 10 µl of 2 mg/ml MWCNT suspension was deposited on the WE area. After each deposition step, the electrode was dried and then stored at room temperature (RT). Nanostructured modified Pt-SPEs were prepared as described in^9^ regarding the electrochemically grown Pt *nanopetals* (NPTs) using the Metrohm Autolab potentiostat PGSTAT 302 N. The bare-SPE was dipped in solutions containing H_2_SO_4_ (95–98%) and Pt salts, H_2_PtCl_6_, and the depositions were carried out by applying −1 V at RT under stirring conditions. A C-electrode was placed in parallel to the Pt-WE as CE, while a Ag-electrode was used as a reference. All bare electrodes were cleaned before the nanostructure formation step by applying +2 V for 60–120 s. Each electrode was then activated before biofunctionalization by acquiring multiple cyclic voltammograms (CVs) between −0.2 V and +1.5 V at 100 mV/s in 0.1 M H_2_SO_4_ until overlapping of two subsequent voltammograms was achieved.

### Redox- p53 product preparation and conformational specific antibody interaction

p53 wild-type recombinant protein was exposed to different pro-oxidant stressors to generate different redox-p53 products, as previously described^23^. In detail, the following pro-oxidant stressors were selected: i) a metal chelator agent (EDTA) that distrains the Zn atom and induces the opening of the protein^24^; ii) a Fenton reaction, mainly mediated by the OH· derived from the decomposition of H_2_O_2_ in the presence of Fe^2+^ and Cu^+^ ^25^ generates a burst of oxygen radicals involved in protein oxidation; iii) a peroxynitrite donor (SIN-1) used to generate *in vitro* nitric oxide (NO) is involved in RNS-related protein modification^18^. Thus, p53 recombinant protein was incubated for 1 h at 37 °C with 200 µM EDTA and 5 mM DTT buffer to generate *denatured p53*. When p53 is exposed to the Fenton reaction (30 μM FeSO_4_ and 10 mM H_2_O_2_), *oxidized p53* is generated, while when 1 mM SIN-1 (Sigma Aldrich) is used, *nitrated p53* is obtained.

In the label-based approach, the anti-p53 conformational specific antibody PAb 240 was used (PAb240, Neomarkers-Lab Vision, Fremont, CA, USA). This antibody recognizes a primary epitope cryptic in the canonical closed conformation (wild-type p53) and is accessible only when p53 protein undergoes conformational changes (denatured p53).

### Sensor characterization

The electroactive surface area was evaluated for every electrode type from the Randles-Sevcik equation by performing CV at a scan rate of 0.1 V/s in a solution containing 1 and 10 mM ferro-/ferri-cyanide ([Fe(CN)6]^3−/4−^), respectively, for C-based and Pt-based electrodes. Indeed, the electrochemical couple Fe^2+^/Fe^3+^ redox process has a very well-known diffusion coefficient (D = 6.20 × 10^−6^ cm^2^)^26^.

Morphological SEM analysis of the different electrode structures was carried out using an FEI XLF30-FEG scanning electron microscope. The accelerating voltage used for the SEM imaging was 2 kV for C-based SPEs and 5 kV for all Pt-based SPEs. Nanostructure imaging was performed directly on the WE area of each SPE.

Effective protein adhesion onto each WE was assessed using fluorescence microscopy. A solution containing 6 µg/ml p53 protein was incubated in the dark for 15 min with a fluorescent labeling reagent (Qubit protein assay organic dye, Invitrogen, Buenos Aires, Argentina), and then it was coated on the electrode surface and incubated overnight at 4 °C. The next day, after a washing step, the fluorescence signal derived from the stained-protein adhesion was acquired with a fluorescence microscope (Olympus IX5, Olympus, Italy).

### SPEs Biofunctionalization

Different electrodes were functionalized according to the protocol previously optimized^23^, as follows. First, for both label-free and label-based approaches, different p53 redox products were immobilized by incubation of a 10 µl drop onto the WE for 2 h at 4 °C. Unspecific drop-casting was adopted for both C and Pt sensors as gold-standard methods. Additionally, considering the different surface chemistry of Pt, another coating approach was performed in parallel on Pt and NPT sensors, adopting the protocol described by Shin *et al*.^27^. Electrodes were incubated with a solution of 100 mM 11-mercaptoundecanoic acid (MUA) in ethanol for 1 h to create the *self-assembling monolayer* (SAM). After the covalent bond between SAM (exposed carboxyl groups) and amine-terminated biomolecules was achieved by dipping electrodes for 30 min in a solution of 50 mM N-ethylcarbodiimide (EDC) and 50 mM N-hydroxysuccinimide (NHS).

For label-based analysis, the biofunctionalization was further completed by the creation of immunocomplexes, including the following steps: i) coating with the specific conformationally altered antibody (PAb 240) for 2 h at RT iii) incubation with alkaline phosphatase (AP)-labeled detection antibody for 1 h 30 min at RT, and iv) incubation in dark conditions for 30 min at RT with the *anodic stripping voltammetry* (ASV) solution to reduce ionic Ag (AgNO_3_) to metallic Ag in the presence of ascorbic acid (AA-p)^28^. For every step, 10 µl of each solution was drop-cast on the WE.

### Electrochemical detection of p53 conformational states

**Figure 1:**
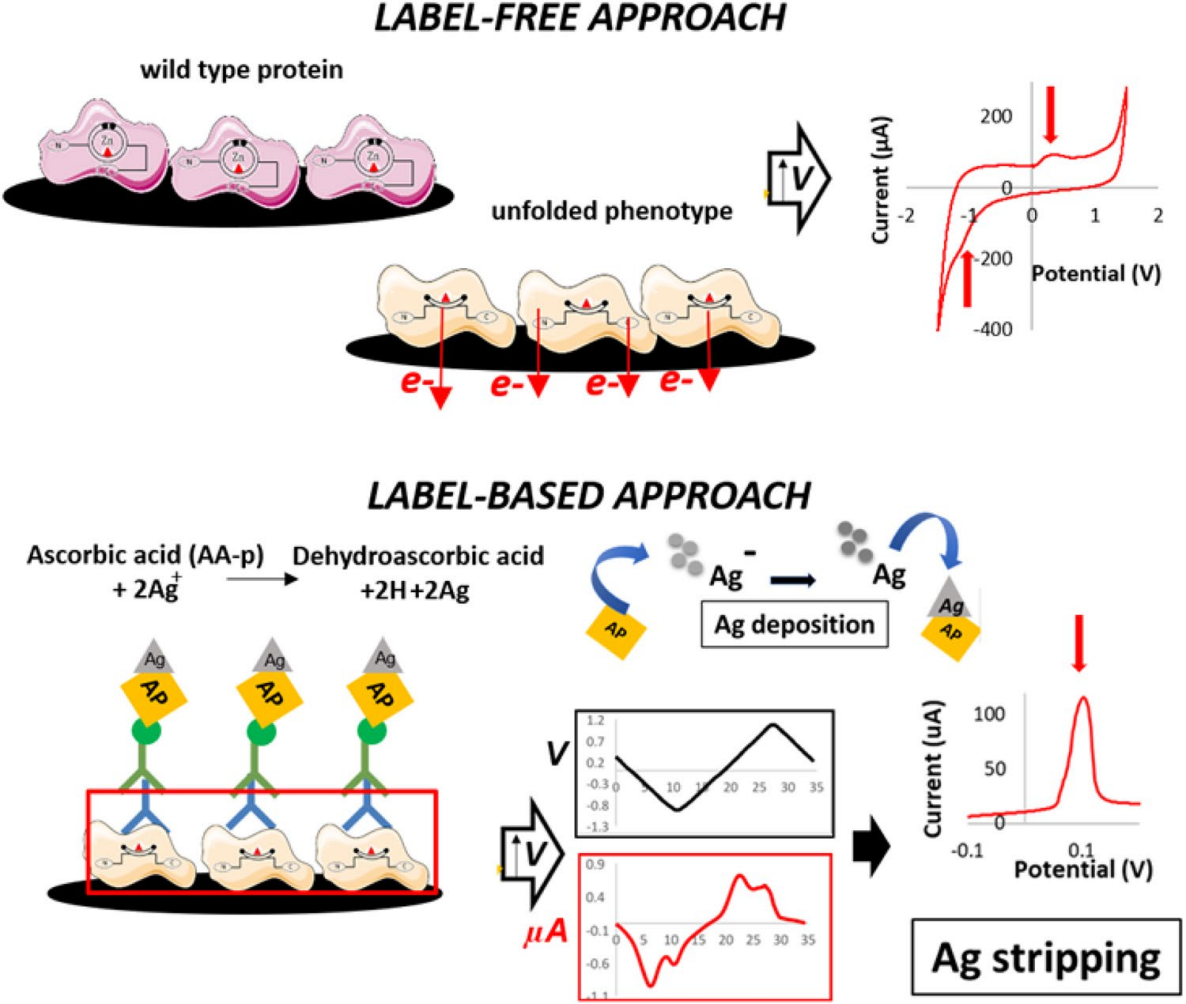
Schematic representation of the label-free and label-based protocols used for protein investigation: The label-free approach analyzed protein directly by means of cyclic voltammetry, while the label-based approach measured indirectly by means of specific antibodies and anodic stripping voltammetry.

Label-free qualitative electrochemical analysis was performed after coating C SPEs WE with different redox-p53 products without immune detection. After an overnight protein coating (6 µg/ml for all the solutions), each sensor was carefully washed using a solution of phosphate-buffered saline (PBS) with 0.5% Tween-20 to remove any unbound protein or other chemicals that could interfere during the measurements. Afterward, the sensors were covered with a 100 µl drop of neutral PBS as the supporting electrolyte, and a CV procedure in the range between −1.6 and +1.6 V, with a scan-rate of 80 mV/s, was performed (Fig. 1). In this potential range, it was possible to record specific peaks due to zinc loss and specific residues exposed during p53 conformation modulation. To ensure that the peaks were in fact due to the protein coating, the CVs obtained from the coated SPEs were compared with those of the nonfunctionalized SPEs covered with 100 µl of PBS.

Direct electron transfer from coated proteins has been investigated by analyzing currents (i_p_) and potentials (E_p_) at four different scan rates (υ) (20, 50, 100 and 200 mV/s) of oxidation/reduction peaks. The reversibility and diffusion/adsorption control of the oxidation/reduction processes were investigated by evaluating the linearity of the relation of E_p_ with respect to ln υ and of i_p_ with respect to $\sqrt{{\rm{\upsilon }}}$ or υ^29^. Laviron’s equation defined in the general expression of the linear potential sweep voltammogram in the case of diffusionless electrochemical systems^30^ was used to estimate the electron transfer coefficient (α) and the standard electron transfer rate constant of the surface reaction (k_s_) values as follows.

1 $${E}_{P}=\,{E}_{0}^{\text{'}}+(\frac{RT}{\alpha nF})[\mathrm{ln}(\frac{RT{k}_{s}}{\alpha nF})-\,\mathrm{ln}\,{\rm{\upsilon }}]\,$$

*n* is the electron transfer number, and ${E}_{0}^{\text{'}}$ is the formal potential.

The E_0_ value can be assumed from the intercept of E_p_ vs. υ plot on the ordinate by extrapolating the line to υ = 0. Once the E_0_ value is known, the values of α and k_s_ were obtained from the slope and the intercept of the graphical representations of Ep vs. lnυ, respectively^31,32^.

Label-based qualitative electrochemical analysis was performed after Ag selective reduction caused by immunocomplex detection. Starting from a potential of −0.12 V a *linear sweep voltammetry* (LSV) procedure was performed in the range −0.12 to +0.7 V, with a scan rate of 40 mV/s. This procedure (Fig. 1), which follows the optimized protocol described in^33^, resulted in oxidative Ag peaks proportional to the amount of protein in the solution tested. For each different conformation tested, three single-use C SPEs were tested to compute the mean and standard deviation of the Ag peak value.

Peaks observed both in CVs and in LSVs were analyzed using Nova 1.11 statistical software and were compared after subtracting the baseline to remove the capacitive current contribution.

### Quantification of denatured p53 on bare and nanostructured sensors

Focusing on *denatured p53*, label-free and label-based quantitative electrochemical analyses were then performed to compare the performances of the different materials. Standard solutions containing different concentrations of *denatured p53* were prepared and coated onto bare and nanostructured C and Pt SPEs (as described in section E), and then current peak heights were obtained for each material at each concentration and analyzed and compared. More specifically, a first calibration was performed considering wider range concentrations [8, 6, 4, 2, 1 μg/ml], comparing the responses of bare and nanostructured SPEs. Afterward, considering the higher performances of nanostructured electrodes, a second calibration considering lower concentrations [2, 1, 0.5, 0.2, 0.1 μg/ml] was performed, comparing only nanostructured SPEs. Each analysis was tested in triplicate to ensure a proper calculation of the mean and standard deviation (σ) of the current peaks for each condition. For each of the two concentration ranges, the limit of detection (LOD) was calculated as 3 times the σ of the blank current peak divided by the slope (m) of the calibration curve, the limit of quantification (LOQ) was calculated as 10σ/m, and the sensitivity was calculated as m of the linearized portion of the calibration curve at lower concentration for every material (Table 1). All results are shown as the mean ± standard deviation.

**Table 1:** Summary of all the significant values from the calibration performed using bare and nanostructured SPEs (LOD and LOQ are expressed in µg/ml, while sensitivities are expressed in µa/(µg/ml)).

		Bare SPEs			Nanostructured SPEs					
		range 1–8 µg/ml			range 0.1–2 µg/ml			range 1–8 µg/ml		
		Sensitivity	LOD	LOQ	Sensitivity	LOD	LOQ	Sensitivity	LOD	LOQ
CATHODIC PEAK	C	2.86 ± 0.83	1.60 ± 0.02	5.33 ± 0.06	15.08 ± 3.33	0.55 ± 0.13	1.83 ± 0.43	14.74 ± 3.57	0.57 ± 0.15	1.9 ± 0.5
	Pt	1.94 ± 0.62	1.85 ± 0.05	6.16 ± 0.16	42.13 ± 7.00	**0.20 **±** 0.02**	1 ± 0.4	10.72 ± 2.6	1.10 ± 0.26	3.66 ± 0.86
	Pt Act	3.79 ± 1.56	**0.90 **±** 0.15**	3 ± 0.5	28.47 ± 6.28	0.23 ± 0.05	0.76 ± 0.17	8 ± 4.26	0.74 ± 0.66	2.46 ± 2.2
ANODIC PEAK	C	2.64 ± 0.61	1.71 ± 0.03	5.7 ± 0.10	20.53 ± 7.79	0.54 ± 0.18	1.8 ± 0.6	19.7 ± 6.88	0.58 ± 0.20	1.93 ± 0.66
	Pt	8.62 ± 2.14	0.86 ± 0.04	2.86 ± 0.13	39.01 ± 5.88	**0.17 **±** 0.06**	0.57 ± 0.2	21.11 ± 3.92	0.29 ± 0.06	0.96 ± 0.2
	Pt Act	14.47 ± 4.23	**0.52 **±** 0.12**	1.73 ± 0.4	38.74 ± 11.19	0.25 ± 0.13	0.83 ± 0.43	14.17 ± 13.2	0.76 ± 1.02	2.53 ± 3.4
ASV PEAK	C	20.90 ± 5.23	1.00 ± 0.30	3.33 ± 1.00	15.31 ± 9.16	0.19 ± 0.07	0.63 ± 0.23	27.01 ± 8.81	0.18 ± 0.07	0.6 ± 0.23
	Pt	8.82 ± 2.85	1.34 ± 0.44	4.46 ± 1.46	29.32 ± 5.86	**0.11 **±** 0.02**	0.33 ± 0.06	21.68 ± 4.79	0.17 ± 0.04	0.57 ± 0.13
	Pt Act	14.71 ± 1.38	**0.52 **±** 0.05**	1.73 ± 0.16	37.83 ± 10.70	0.18 ± 0.06	0.56 ± 0.3	20.64 ± 1.51	0.35 ± 0.03	1.16 ± 0.1

## Results

### Electrochemical characterization

**Figure 2:**
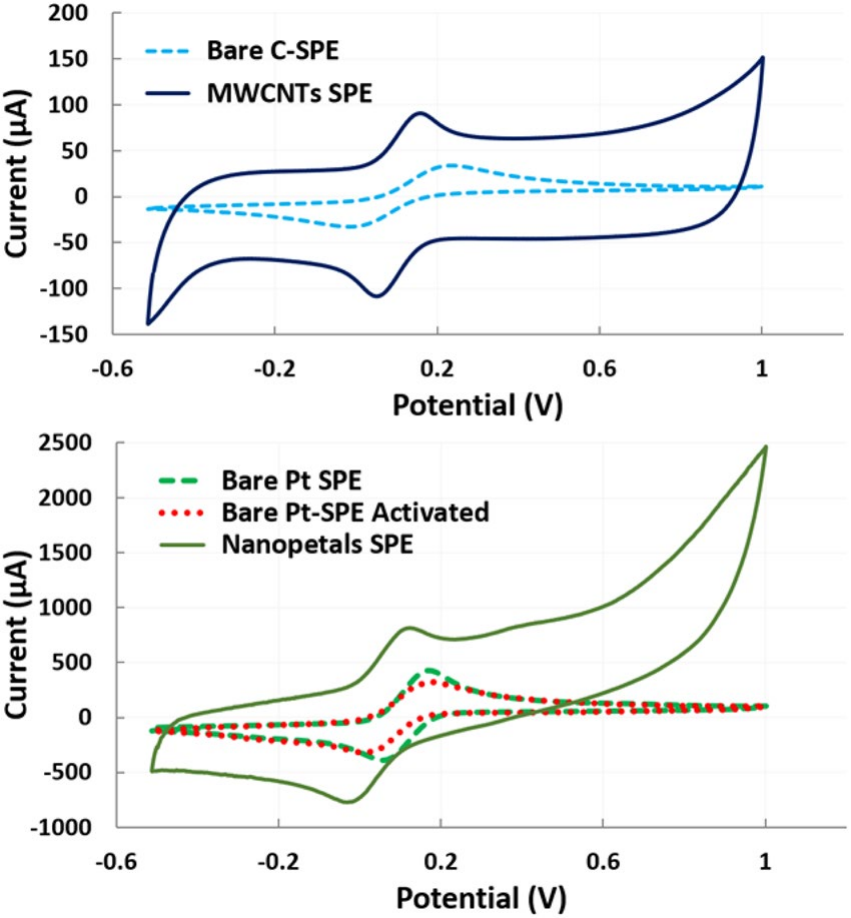
Ferro-cyanide analysis for electroactive area evaluation: above, C-SPE and MWCNT-modified C-SPE reaction; below, Pt-SPE, nanopetal-structured Pt-SPE and activated Pt-SPE comparison.

From the ferro-/ferri-cyanide analysis summarized in Fig. 2, it is highly apparent that the current enhancement due to nanostructures on the bare SPE WE (MWCNTs and NPTs deposited on C and Pt SPEs, respectively).

Indeed, it was found that the electroactive area was increased by 37.2 ± 10.9 cm^2^ and 53.3 ± 1.3 cm^2^ relative to those of the C-bare and Pt-bare SPE WE after modification with MWCNTs and NPTs, respectively. The high variability linked with the increase of the area for MWCNTs is the consequence of a reduced control on their deposition due to the drop-casting technique.

**Figure 3:**
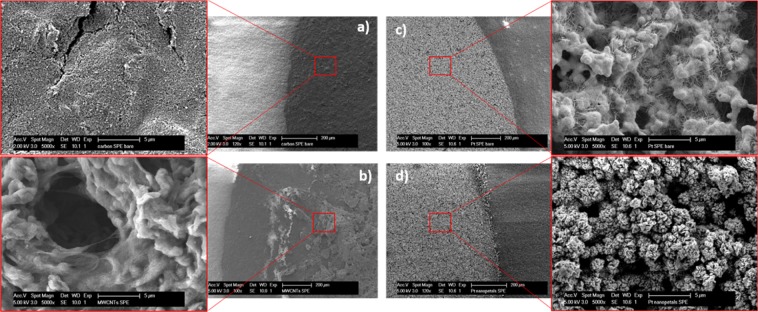
Characterization of bare and nanostructured electrodes using SEM: in detail, carbon SPEs (**a**), drop-casted MWCNTs on C SPEs (**b**), platinum SPEs (**c**) and electrochemically grown Pt NPTs on Pt SPEs (**d**).

On the other hand, electrochemically deposited NPTs ensured a more controllable and reproducible approach for nanostructuring the WE, as confirmed by the low variability observed in the electrochemical characterization (Fig. 2). SEM analysis of the WE areas revealed the SPE morphological characteristics, in agreement with the results from ferro-/ferri-cyanide characterization (Fig. 3). Regarding C-SPEs (Fig. 3a), the bare electrode was characterized by a rough and nonhomogeneous surface due to its manufacturing process. By drop-casting MWCNTs onto the WE surface (Fig. 3b), an increase in the active area observed with the electrochemical characterization was observed, in agreement with the morphology revealed by the SEM analysis. Thus, the effectively immobilized clusters of MWCNTs, randomly oriented on the WE surfaces, increase the surface-to-volume ratio of the electrode available for electron exchange. Regarding the bare Pt electrode, a peculiar morphology with a micrometer rugosity can be observed, attributed to the platinum microparticles held together with a polymeric formulation in Pt-SPEs (Fig. 3c). In comparison, the surface-to-volume ratio of Pt WE strongly increases when nanostructured with NPTs (Fig. 3d). Furthermore, SEM images of electrode borders highlight more defined borders using electrochemical nanodeposition rather than using a drop-casting approach.

**Figure 4:**
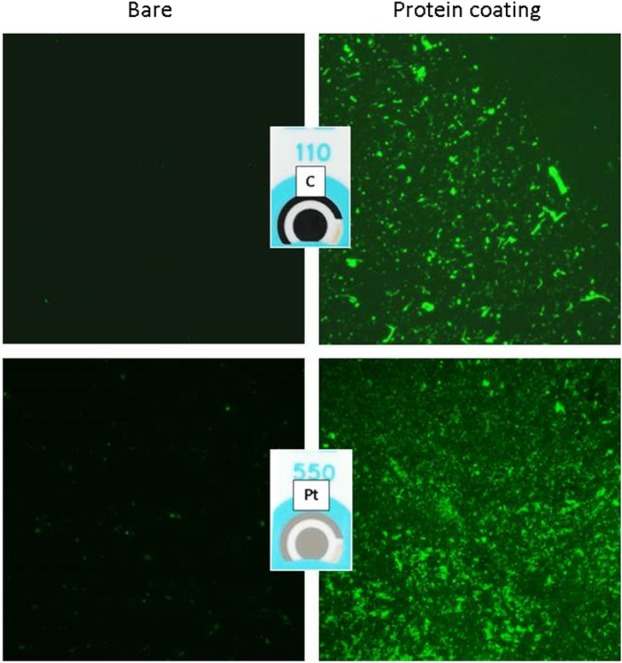
Characterization of protein adhesion on WE by using fluorescence microscopy. Representative pictures of electrodes coated with fluorescently labeled p53 protein (right) compared to bare materials (left), both acquired with a 10X magnification on C (top) and Pt (bottom).

The effective protein adhesion onto both C and Pt WEs was also confirmed by fluorescence microscopy when the electrodes coated with fluorescently labeled p53 recombinant protein were compared with the bare one (Fig. 4).

### Electrochemical detection of different p53 redox products

#### Label-free analysis

**Figure 5:**
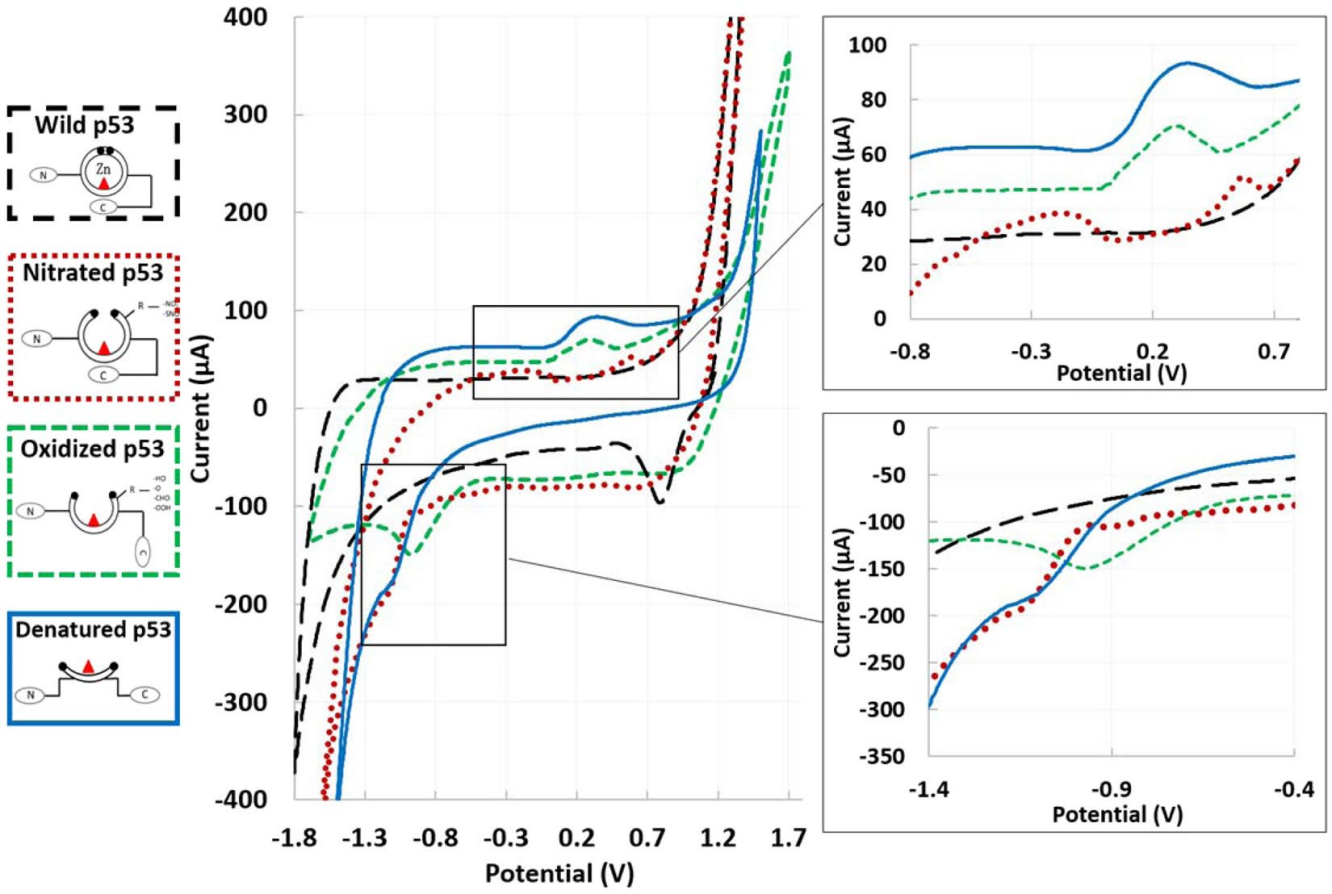
Electrochemical detection of different p53 redox products. On the left, label-free approach, a comparison between CVs performed coating SPEs with *wild-type (black)*, *nitrated (orange)*; *oxidized (green)* and *denatured (blu*e*) p53*. The amino acid residues available for oxidative/nitrosative modifications are represented as R.

CV results from label-free electrochemical detection of different p53 redox products are shown in Fig. 5. Interestingly, specific electrochemical peaks could be correlated to each different p53 conformation. In detail, the denatured-open isoform of p53 protein shows two specific peaks: i) a cathodic peak at approximately +0.4 V and ii) an anodic peak at approximately −1 V. The cathodic peak suggests a contribution of specific amino acids (tyrosine and cysteine) exposed during unfolding, while the anodic peak suggests a contribution of the zinc atom released during the unfolding process within the potential window [−0.7, −1.1 V]. In contrast, the oxidized conformation shows smoother redox peaks, probably due to a partially denatured structure, with both peaks both shifted towards lower absolute potentials (cathodic approximately 0.3 V and anodic at −0.9 V). Finally, in the nitrated conformation, both cathodic and anodic peaks appear at approximately the same potentials as the peaks of the denatured p53, but each one split into two peaks: two cathodic peaks were observed at approximately +0.6 V and −0.2 V, and two anodic peaks were observed at −0.8 and −1 V. Since all the different p53 redox processes were estimated using the same substrate material (C-SPEs), these shifts in the peak potentials have to be attributed to differences in the protein conformations and in the different functional groups added during nitration and oxidation.

**Figure 6:**
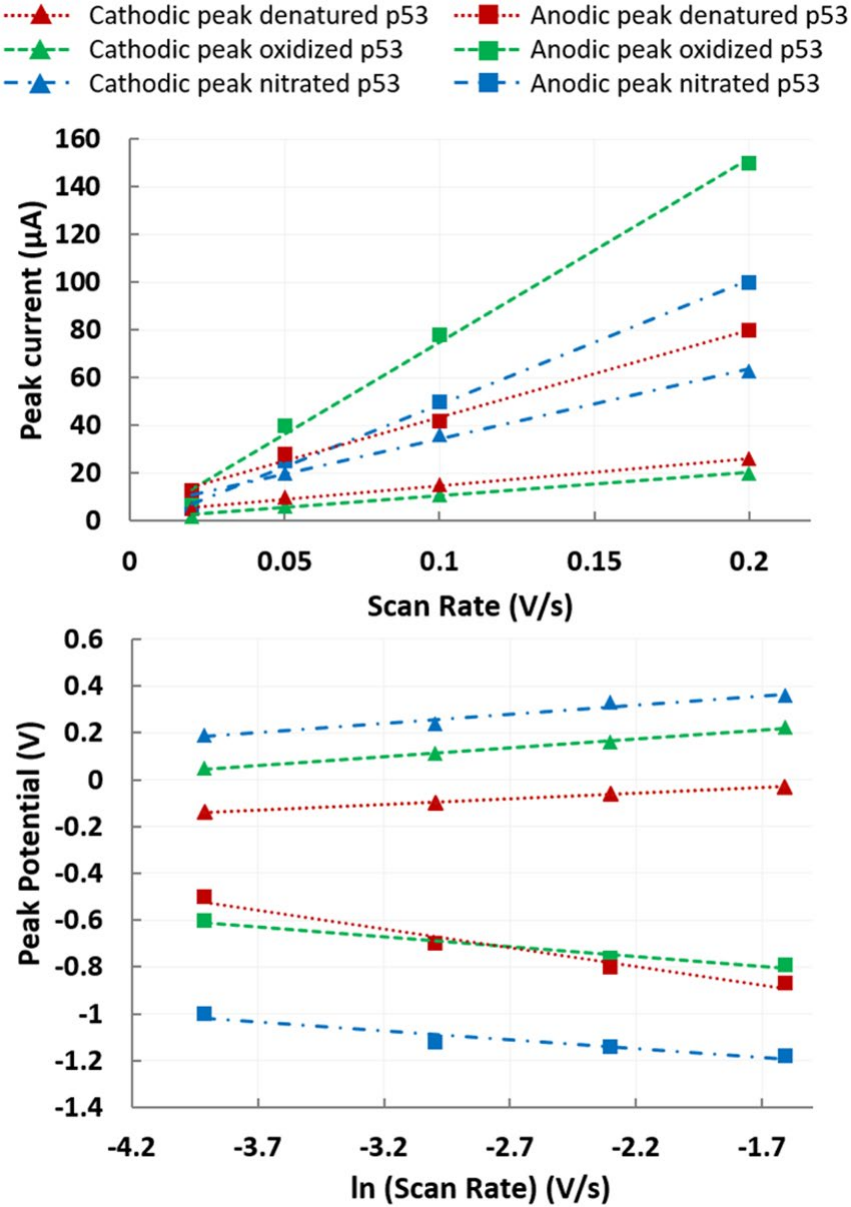
Label-free analysis of the peaks: Plots evaluating the linearity of the relationship between current peak and scan rate and between peak potential and natural logarithm of the scan rate, confirming protein adsorption and the irreversibility of the reaction.

The linearity observed between i_p_ and υ (Fig. 6) suggests that the electron transfer occurs from an adsorbed layer of proteins through a surface-controlled electrochemical process^34^. Furthermore, a linear relation between E_p_ and log(υ) can be observed, with ΔEp increasing with increasing υ. The value of ΔEp for all the different conformations appears to be significantly higher than 59 mV, above which only pseudoreversible or irreversible process can take place. Furthermore, the ratio between the cathodic and anodic absolute current values appears different from 1 for all conformations. Considering the above findings and the higher probability of correlating the anodic (E_a_) and cathodic (E_c_) peaks with a different source (E_a_ with the zinc atom and E_c_ with electroactive amino acids) rather than with a single redox process, we analyzed each peak as an individual irreversible peak, according to Laviron’s theory. The E_0_ values for each peak could be deduced from the intercept of a plot of E_p_ vs. υ on the ordinate by extrapolating the line to υ = 0 (Table 2). Starting from E_0_ and from the graphical representations of E_p_ vs. lnυ, the values of αn and k_s_ were obtained from the slope and intercept, respectively (Fig. 6). Since for a totally irreversible electron transfer, α was assumed to be 0.5, n values could be calculated, suggesting that one electron was involved in the oxidation/reaction. The values of k_s_ for all the conformations are on the order of 1 s^−1^, with lower values for the cathodic peaks than for the anodic peaks for all the conformations, suggesting a faster electron exchange for the anodic peaks.

**Table 2:** Summary of α·n and k_s_ values calculated using Laviron’s theory for nonreversible reactions.

	Cathodic peaks		Anodic peaks	
	α·n	k_s_ (s^−1^)	α·n	k_s_ (s^−1^)
Denatured p53	0.32	0.33	0.32	1.35
Oxidized p53	0.33	0.35	0.30	2.04
Nitrated p53	0.52	0.58	0.30	1.83

#### Label-based analysis

**Figure 7:**
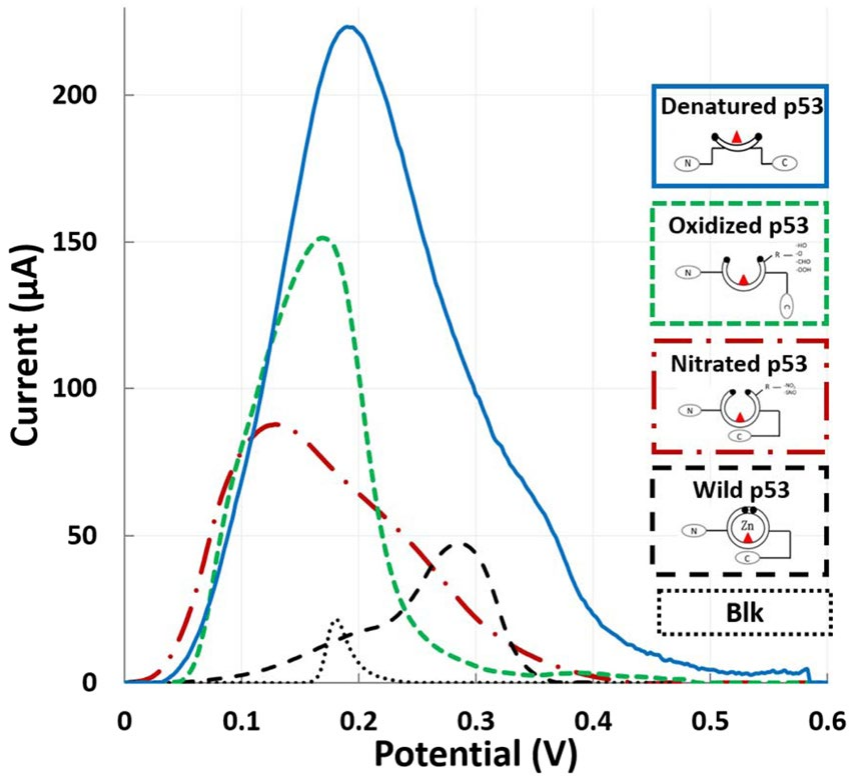
Label-based qualitative detection of different p53 redox products using silver stripping voltammetry. Peaks resulting from protein quantification by the well-known conformationally altered antibody Ab240 are represented.

The results obtained from label-based analysis of different p53 redox products confirmed a higher specificity of PAb 240 for the open isoform (*denatured p53*), while no affinity for the wild-type conformation was found, as reported in Fig. 6. Interestingly, PAb 240 recognizes *denatured*, *oxidized* and *nitrated p53* with decreasing affinity, suggesting that the origin of oxidant species (RNS or ROS) and the rate of oxidant for the reaction could give rise to different p53 redox products. In addition to the main peak due to Ag stripping, secondary peaks were observed. More specifically, wild-type p53 shows an additional peak at approximately +0.1 V. *denatured p53* and *oxidized p53* instead show an additional peak at approximately +0.4 V, while *nitrated p53* shows an additional peak at approximately +0.3 V. A background signal due to unspecific Ag deposition and wild-type p53 misrecognition could be observed (Fig. 7).

### Quantification of denatured p53 on bare and nanostructured sensors

#### Label-Free Analysis

**Figure 8:**
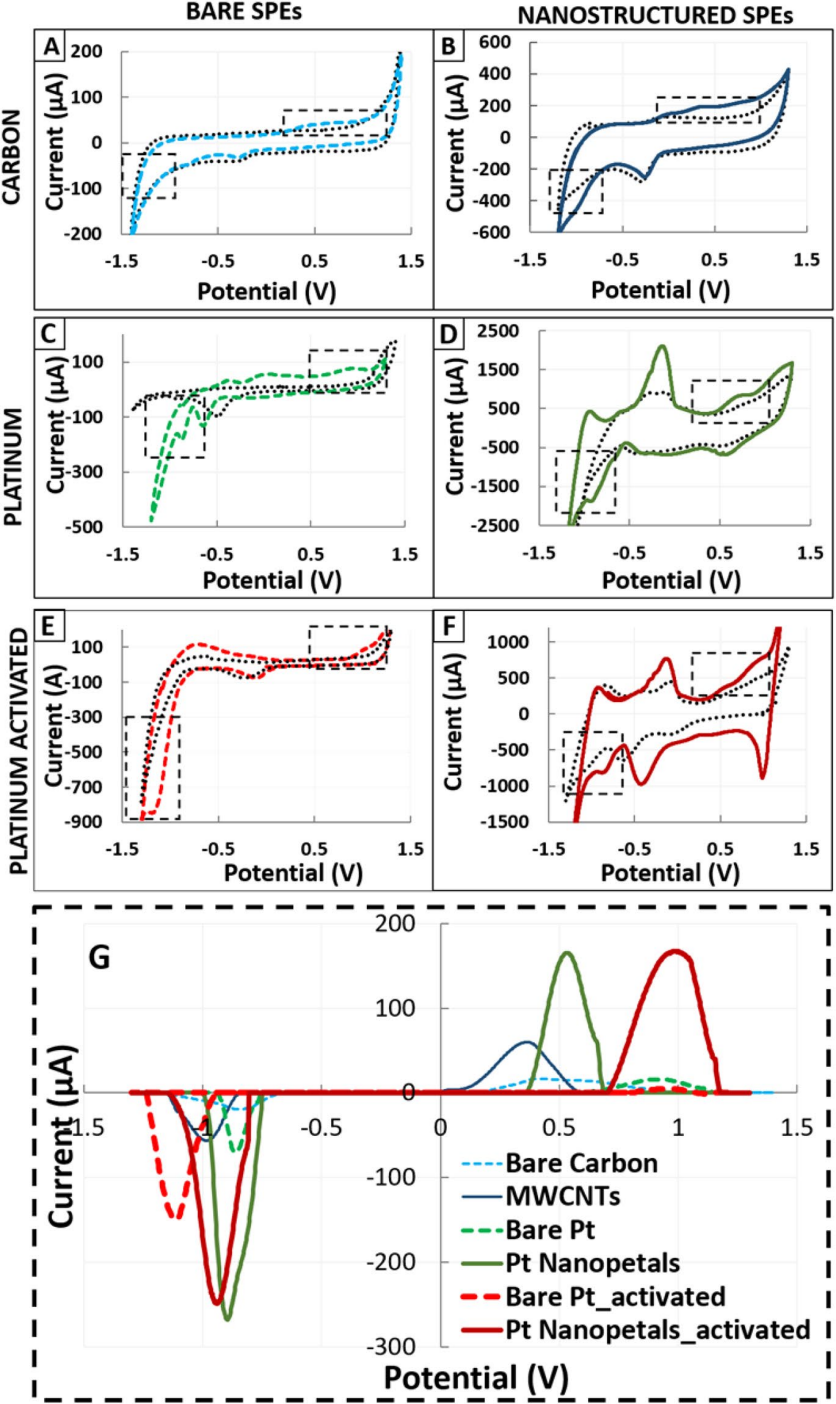
(**A–F**) CVs of control SPEs and SPEs coated with denatured p53, with and without nanostructures. (**A**) C vs (**B**) MWCNTs, (**C**) Pt vs (**D**) Pt NPTs act, (**E**) Pt vs (**F**) Pt NPTs), (**G**). Comparison between peaks highlighted in fig. (**A**–**C**), enhanced subtracting the baseline from the signal, to compare the effective contribute given by the same concentration of denatured p53 coated on different materials.

The significantly increased peak heights and reduced peak potentials observed for both MWCNT- and NPT-modified electrodes, with respect to the corresponding bare SPEs, suggested the significant role of nanostructures in improving the sensitivity in the quantification of denatured p53 detection (Fig. 8). The nanostructures maintained a similar enhancement in terms of peak height for both cathodic and anodic peaks. In addition, for bare sensors, chemical adsorption shows a significant improvement compared with physical adsorption. Since only denatured p53 has been selected for testing with this approach, all the differences in terms of peak heights and potential are attributed exclusively to the different materials and their nanostructures.

**Figure 9:**
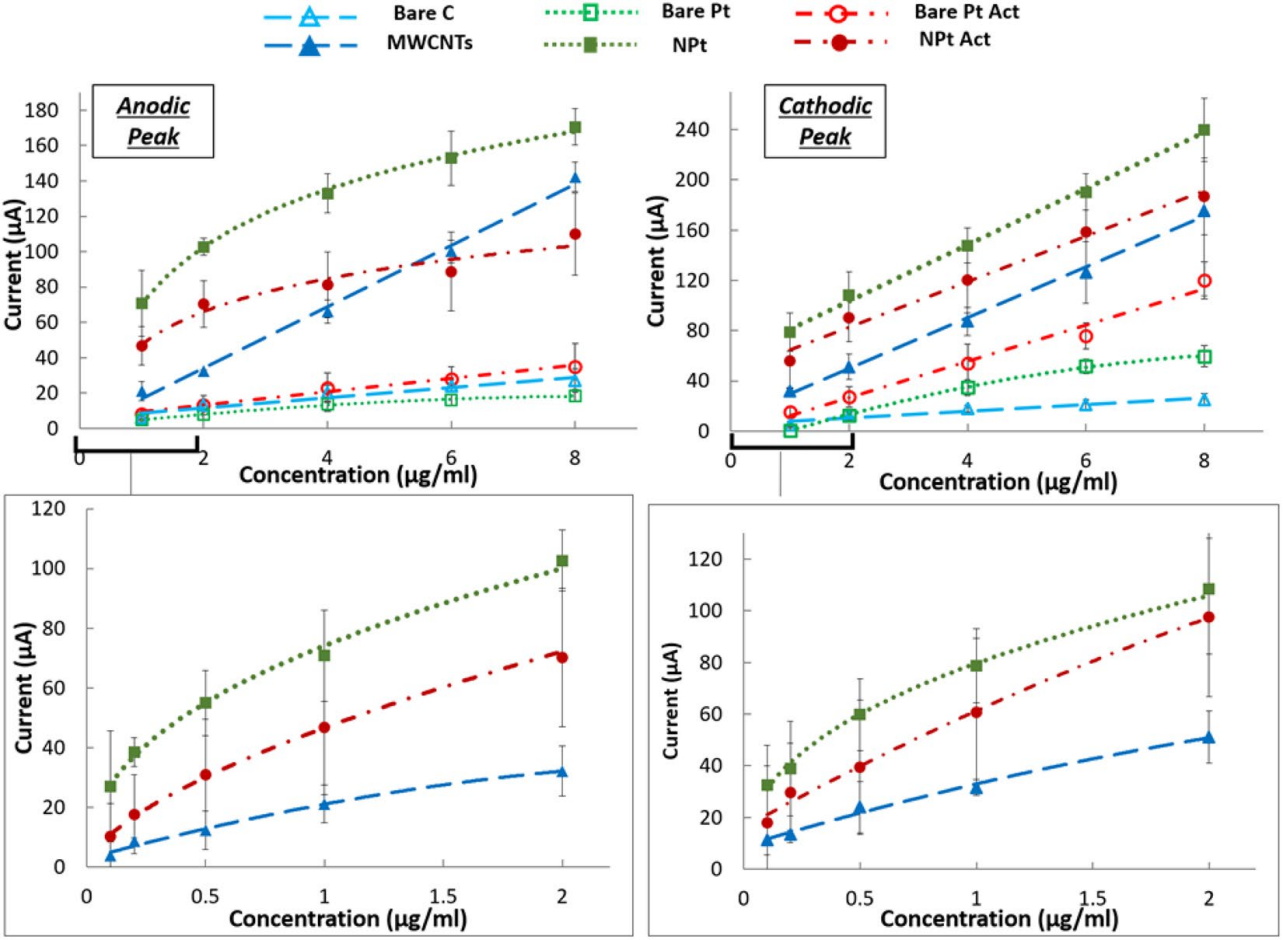
Label-free calibration of denatured p53 using CV on SPEs of different materials. The calibration plots compare bare and nanostructured C- and Pt-based materials. For Pt, physical and chemical adsorption (Pt Act) are compared.

Table 1 summarizes the results obtained in terms of LOD, LOQ and sensitivity for the different materials tested. These parameters are graphically shown by comparison among the calibration plots in Fig. 9, which highlights an improved LOD for all the nanostructured electrodes compared to the corresponding bare form. The LOD obtained for p53 covalent chemical absorption is improved by 1.5-fold compared with unspecific physical coating (Table 1) only for bare Pt. The same activation performed on the nanostructured sensor does not show the same increase. This specific finding suggests that NPTs can improve the protein coating effectiveness by increasing the roughness of the electrode surface. Overall, for bare SPEs, the lowest LOD was obtained for activated Pt SPEs (0.90 ± 0.15 µg/ml for the cathodic and 0.52 ± 0.12 µg/ml for the anodic peak). In contrast, for the nanostructured SPEs, the lowest LOD was obtained for physically coated NPT-modified SPEs (0.20 ± 0.02 µg/ml for the cathodic and 0.17 ± 0.06 µg/ml for the anodic peak).

Regarding the reproducibility and repeatability of the measurements, which were evaluated as a coefficient of variation (*standard_dev/average*) for each quantification, greater variability could be observed for those performed on nanostructured C-SPE with chemical adsorption (relative standard deviation of ~30%), while the best results were observed for bare and electrochemically modified NPT electrodes (relative standard deviation <20%). Both the MWCNT electrodes and the EDC-NHS Pt-activated electrodes showed greater variability between the measurements due to the manual drop-casting technique in the first case and the lack of standardization of the self-assembled monolayer in the second case. Despite this limitation, MWCNTs improved the LOD by 2-fold compared to bare C SPEs. In contrast, NHS-EDC applied to nanostructures showed a worse result than the non-activated nanostructured Pt sensors, in terms of both LOD and sensitivity (Table 1).

#### Label-Based Analysis

**Figure 10:**
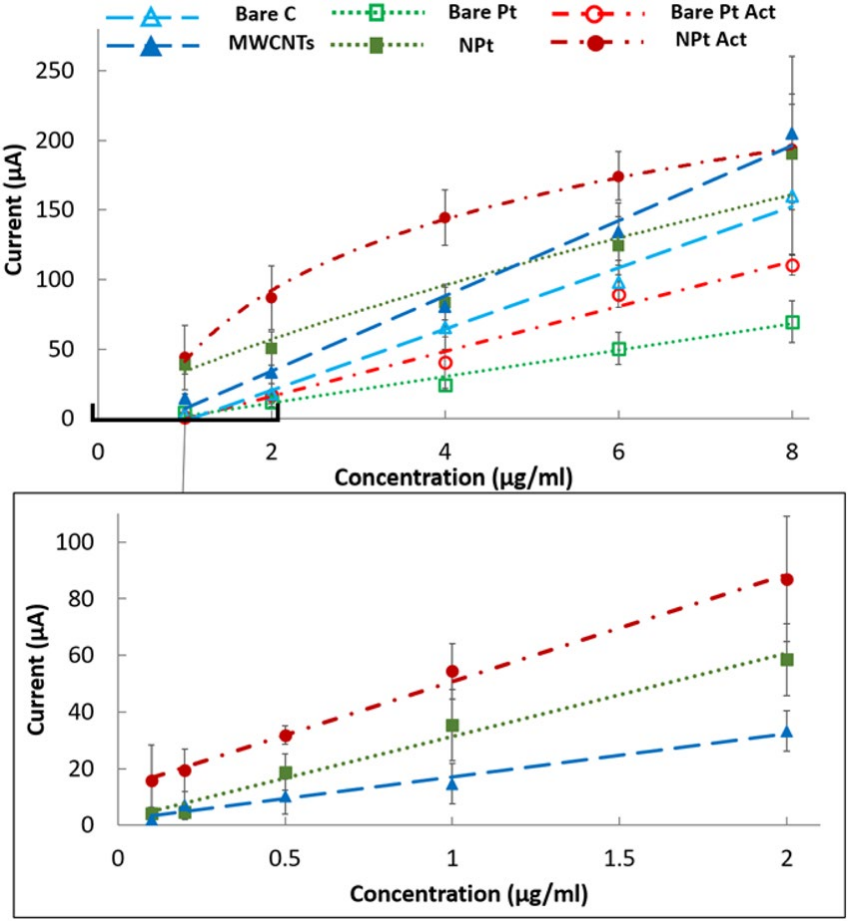
Label-based calibration of denatured p53 performed using the ASV protocol with PAb240 on SPEs of different materials. The plots show the calibration comparing bare and nanostructured C- and Pt-based materials. For Pt, physical and chemical adsorption (Pt Act) are compared.

The results obtained from the calibration performed on different materials are summarized in Fig. 10 and Table 1.

In agreement with the label-free analysis, the lowest LOD was obtained from activated Pt SPEs among bare SPEs (0.52 ± 0.05 µg/ml) and from physical adsorption on Pt NPTs SPEs among nanostructures (0.110 ± 0.02 µg/ml).

Thus, the chemical absorption improved the LOD only for bare Pt sensors (from 1.37 µg/ml to 0.52 µg/ml) but not for NPTs Pt sensors, where the physical absorption showed the best result (110 ng/ml compared to 180 ng/ml). Overall, nanostructured sensors, both C and Pt, contributed to improving the LOD compared to the bare materials, as highlighted in Table 1. Comparing the performances of the different materials, opposite behaviors were observed in bare and in nanostructured SPEs. Specifically, C showed better performance in the bare SPEs, while Pt showed better performance in the nanostructured ones. Furthermore, the variability is greater for MWCNTs than for NPTs, confirming that electrochemical deposition is a more reliable method than drop-casting, in agreement with the label-free analysis.

## Discussion

The use of electrochemistry to directly investigate protein conformation has been extensively highlighted due to the possibility of detecting intrinsic redox reactivity when specific potentials are applied^20,35^. The electroactivity of the metalloprotein p53 can be explained by the presence of redox-sensitive thiol groups (-SH) in the two clusters of cysteines (Cys) in the DNA-binding domain. Mutation of these Zn^2+^ ligands diminishes the sequence-specific DNA binding of p53, and it cannot be excluded that redox modification might induce conformational changes affecting the stability of p53^36^.

In contrast to previous studies on p53^20,21^, we performed a comprehensive electrochemical study investigating different p53 conformations in the transition from wild type to an open isoform. Electrochemical peaks correlated with the different protein conformations appear supported by the electroactive behavior of specific amino acids, as highlighted in the literature.

In the qualitative analysis performed with the *label-free* approach, the enhancement of the peaks due to protein unfolding appears in perfect agreement with the results of recent studies related to the electrochemistry of nonconjugated proteins and glycoproteins^37^. The cathodic peak at +0.4 V observed for the completely opened conformation could be explained by the presence of abundant tyrosine and cysteine in the inner part of the protein, which become exposed due to the unfolding process, in agreement with previous works investigating amino acid electrochemical behavior on C and Pt substrates^38–40^. Furthermore, the high amount of arginine within the DNA binding domain might contribute to the oxidative/reductive peak at approximately 0.5 V^41^. Regarding the anodic peak at −1.0 V, even with different electrochemical techniques and electrode materials, our findings confirm the hypothesis presented in^20^, where the peak was correlated with electrocatalysis from the structurally affected zinc-binding region. The attenuated peaks observed in the *nitrated* and *oxidized* conditions could be explained considering the partially opened conformation and the exposure of nitrated tyrosine and cysteine, as supported by previous investigations of their electrochemical behavior^42^. In particular, for oxidized p53, the peak redox potential shifted towards lower absolute values, which could be attributed to tyrosine oxidation, as demonstrated in^1^. In the nitrated p53, the shift of the cathodic peak towards higher potentials and the anodic peak at −0.8 V agree with the results presented in^43^. Here, the addition of a nitro group was shown to result in a shift of the oxidation peak potential of approximately 150 mV and in an additional reduction peak at the potential of −0.75 V.

Considering the translation of label-free qualitative detection into clinical practice, possible interfering agents due to human blood protein components need to be discussed. The most abundant of these endogenous proteins are albumin, presenting an oxidative/reductive peak at approximately 0.7–0.8 V on C electrodes^44^, and globulin, which not highly electroactive but sometimes used to enhance electrochemical detection^45^. In light of these, it is reasonable to state that the different contributions due to possible interfering substances are identifiable and can be separated from peaks due to different p53 conformations by adopting an appropriate data processing technique^46^.

In the label-based approach applied to recognize the different p53 redox products, the higher specificity of PAb240 for *denatured* p53 was in agreement with the literature^18^. Interestingly, the subsequent decreasing affinity for *oxidized* and *nitrated* p53 could be due to a putative different exposure of the epitope recognized by PAb240. Therefore, the different affinity of this antibody in recognizing p53 redox products suggests the possibility of distinguishing different p53 conformational states during the transition from the wild-type form to the unfolding structure. Moreover, some discrepancies can be found between the *label-based* ASV results presented here and the results obtained in previous work, where IL-8 was the protein target to be quantified^33^. Using this conformational-altered antibody, multiple embedded peaks can be observed, probably due to an intrinsic redox reactivity of the p53 protein even after Ag chemical deposition. In detail, the electrochemical peak due to antibody exposure might overlap with the peak from Ag stripping and thus result in a composite peak. These additional peaks are noticed at a potential of nearly +0.4 V for denatured p53 and approximately +0.1 V for the oxidized and wild-type p53, as highlighted in Fig. 8. All these data are consistent with the results obtained from *label-free* analysis and hence are explained by the possible contribution of cysteine groups exposed after inducing the opening of the protein^47,48^ and by the influence of proline^49^.

Regarding the comparison between different materials when detecting *denatured p53*, the differences obtained in terms of peak height and redox potentials can be explained by three main factors: i) formation of nanostructures, ii) the bulk material and iii) the protocol used for protein coating. Regarding the material, the positive shift registered in the *denatured p53* cathodic peak using Pt appears to agree with those observed in the electrochemistry of cysteine^50,51^, where the use of Pt instead of C introduced a shift in the cathodic peak of cysteine, increasing the max current to approximately +0.9 V instead of +0.5 The shift of −250 mV observed when both C and Pt are nanostructured could be explained by the enhancement of the redox reaction introduced by nanostructure formation. This is in agreement with the literature regarding both C vs MWCNTs^52^ and Pt vs Pt NPTs^53^.

The calibrations performed for *denatured p53* quantification showed quite linear trends for the bare electrodes, while a logarithmic trend was observed for nanostructured electrodes, with a higher sensitivity observed in the lower concentration range (100 ng/ml to 1 µg/ml), where the curve can be considered linear. This appears consistent with previous data obtained using MWCNTs^54^ and Pt NPTs^55^. The results obtained using nanostructures showed a significant enhancement in terms of LOD, with a decrease of 5-fold using MWCNTs and 13-fold using Pt NPTs compared to their respective bare electrodes. The lowest LOD (110 ± 20 ng/ml) obtained with Pt NPTs is also in agreement with^56^ and might be explained by considering both the higher protein affinity of Pt-based nanomaterials^57^ and the increased available surface for p53 coating obtained with the electrodeposition technique. Thus, Pt NPT nanostructure formation appeared to be the most promising technique, even considering the lower variability observed when compared with MWCNT nanostructured electrodes^17^. Although the LODs are still one order of magnitude higher than that achieved using immune-detection techniques (e.g., ELISA) for protein quantification^58^, the strength and the novelty of the results obtained are attributed to the easily reproducible method and the possibility to obtain conformational information.

Additionally, the agreement of the label-free outcomes with the label-based outcomes indicates that this is a promising approach to exploit the intrinsic redox activity of proteins even without the use of expensive labels, as reported mainly for p53 protein detection^56,59^.

## Conclusion

A conformational investigation of the p53 protein based on two easily reproducible electrochemical methods (CV and ASV) using screen-printed nanostructured sensors is here presented. In contrast to other studies addressing this purpose, the combination of label-free and label-based approaches allowed us to collect both qualitative and quantitative information about four different p53 redox products during the transition from the wild-type to the denatured-open isoform.

Interestingly, the label-free analysis identifies specific peaks correlated with *wild-type, oxidized, nitrated and denatured p53*. These different conformations could be discriminated thanks to specific cathodic/anodic electrochemical peaks located at +0.4 V/−1 V for denaturation, +0.3 V/−0.9 V for oxidization, and +0.6 and −0.2 V/−1 and −0.8 V for the nitrated conformation. The values of electron transfer k_s_ were on the order of 1 s^−1^, with a difference between anodic and cathodic peaks, suggesting higher electron transfer for anodic peaks. Label-based analysis confirmed and reinforced these results, taking advantage of antibody*-*antigen specificity, recognizing with decreasing affinity the *denatured*, *oxidized* and *nitrated p53*.

Moreover, aiming at the possibility of adopting this method not only for the detection of the specific conformation but also for its quantification, the results obtained by the quantification of *denatured* p53 indicate that this is an attractive approach, with the possibility of future integration in portable devices. Indeed, the use of nanostructures showed a significant enhancement in terms of LOD (up to 7-fold) and sensitivity (up to 3-fold) compared with bare sensors. The best performances among the bare SPEs were obtained for Pt-activated electrodes (LOD = 520 ng/ml, with both approaches). By introducing nanostructures, this LOD could be reduced by 5-fold, with the lowest value of 110 ng/ml obtained for non-activated electrochemical growth of Pt NPT via the label-based method.

Overall, the results obtained demonstrated the ability of our qualitative analysis to discriminate between different p53 redox products and the strength of our quantitative analysis to perform a calibration of *denatured p53* on different nanostructured electrodes. Thus, the time and cost-effective methods presented here, which combine powerful nanostructures with electrochemistry and are based on easy and reproducible techniques, can be considered a first step towards the realization of complex automated devices for clinical practices (e.g., point of care, lab on a chip).
